# Pan‐TRK expression and *NTRK* gene aberrations in meningiomas: association with tumor grade and proliferative activity

**DOI:** 10.1002/2056-4538.70105

**Published:** 2026-07-01

**Authors:** Yinan Zhu, Mingfang Sun, Wanchen Lu, Ziyue Wang, Haiyan Xi, Yan Song, Hongtao Xu, Xuyong Lin

**Affiliations:** ^1^ Department of Pathology The First Hospital of China Medical University Shenyang PR China; ^2^ Department of Pathology College of Basic Medical Sciences of China Medical University Shenyang PR China; ^3^ Department of Pathology National Cancer Center/National Clinical Research Center for Cancer/Cancer Hospital, Chinese Academy of Medical Sciences and Peking Union Medical College Beijing PR China

**Keywords:** meningioma, pan‐TRK, NTRK rearrangement, immunohistochemistry, FISH, clinicopathological correlation

## Abstract

Tropomyosin receptor kinase (TRK) fusions are actionable oncogenic drivers, and pan‐TRK immunohistochemistry (IHC) serves as a reliable screening tool for *NTRK gene* aberrations. However, the expression profile and clinical significance of pan‐TRK in meningiomas remain unclear. This study aimed to characterize pan‐TRK expression, its correlation with clinicopathological features, and underlying NTRK rearrangement status in meningiomas. We retrospectively analyzed 70 primary intracranial tumor specimens, including 50 meningiomas (21 WHO grade 1, 26 grade 2, and 3 grade 3) and 20 non‐meningioma CNS tumors (9 solitary fibrous tumors, 6 hemangioblastomas, and 5 schwannomas) from the First Affiliated Hospital of China Medical University (2020–2022). Pan‐TRK IHC was performed using the Ventana EPR17341 antibody, with fluorescence *in situ* hybridization (FISH) validating *NTRK gene* aberrations in positive cases. Clinicopathological correlations were analyzed using chi‐square/Fisher's exact tests and Spearman's rank correlation. Pan‐TRK immunoreactivity was detected in 12/50 (24.0%) meningiomas, predominantly with cytoplasmic staining (75.0%). Positivity was significantly higher in high‐grade (WHO 2/3) meningiomas (7/29, 46.7% versus 3/21, 14.3% in grade 1; *p* = 0.031) and tumors with Ki‐67 index ≥5% (7/15, 46.7% versus 5/35, 14.3% in Ki‐67 <5%; *p* = 0.027). No pan‐TRK expression was observed in non‐meningioma tumors (0/20). FISH confirmed *NTRK gene* aberrations in 2/12 (16.7%) pan‐TRK‐positive cases (both WHO 3 anaplastic meningiomas), with strong/moderate IHC staining correlating with aberrations. Pan‐TRK is frequently expressed in meningiomas, particularly in high‐grade and proliferative tumors, and may have preliminary utility in differentiating meningiomas from other CNS tumors. However, *NTRK gene* aberrations are rare, necessitating FISH or next‐generation sequencing confirmation for pan‐TRK‐positive cases to identify candidates for TRK‐targeted therapy. Further studies are needed to clarify the biological role of non‐fusion‐mediated pan‐TRK overexpression in meningioma progression.

## Introduction

Tropomyosin receptor kinase (TRK) is a family of neurotrophic factor receptor proteins, encompassing three highly homologous isoforms: TRKA, TRKB, and TRKC. These receptors are encoded by the neurotrophic tyrosine receptor kinase (NTRK) genes NTRK1, NTRK2, and NTRK3, respectively [1, 2, 3]. Under physiological conditions, TRK family proteins are predominantly expressed in neural tissues, where they modulate neuronal differentiation, survival, axonogenesis, and dendritogenesis. They play pivotal roles in embryonic development and the maintenance of normal nervous system homeostasis [4]. When NTRK genes undergo fusion with partner genes, the resultant aberrant TRK fusion proteins constitutively activate downstream signaling cascades in a ligand‐independent fashion, thereby driving tumor cell proliferation and metastasis. NTRK fusions have been identified in various solid tumors, including those of the lung, gastrointestinal tract, thyroid gland, primary brain, and soft tissues [5].

Given that NTRK overexpression promotes tumor initiation and progression, malignant tumors harboring NTRK aberrations may derive clinical benefit from TRK inhibitors. Several TRK inhibitors, such as larotrectinib and entrectinib, have been approved by the U.S. Food and Drug Administration (FDA) [6, 7]. A study by Doz *et al*. demonstrated that larotrectinib induces rapid and durable responses in TRK fusion‐positive primary central nervous system (CNS) tumors [8]. Thus, detecting pathogenic NTRK fusions confers substantial clinical significance. Immunohistochemistry (IHC) is a rapid, cost‐effective, and widely used pathological technique for evaluating protein expression in tumor cells. The pan‐TRK antibody simultaneously detects the overexpression of TRKA/B/C proteins. With a sensitivity of 95.2% and specificity of 100% for transcriptional NTRK fusions, pan‐TRK IHC serves as a reliable surrogate marker for routine screening of NTRK gene fusions [9].

Meningiomas are the most prevalent primary intracranial neoplasms, accounting for approximately 37.6% of all CNS tumors. They predominantly occur in elderly individuals, with increasing incidence and a higher proportion of aggressive subtypes in patients aged ≥65 years [10, 11]. Surgical resection achieves curative outcomes in 70–80% of cases, while incomplete resection is the primary risk factor for recurrence [12, 13]. However, tumor location may limit surgical radicality – for instance, in cases of brain parenchyma invasion or vascular involvement. Additionally, neurosurgical procedures can lead to neurological, neurocognitive, and functional sequelae, compromising patients' quality of life [10]. Elderly patients undergoing meningioma surgery have significantly prolonged hospital stays and an elevated risk of complications such as pneumonia and pulmonary embolism [14]. A 25‐year long‐term follow‐up study reported 10‐year and 25‐year recurrence rates of 13% and 38%, respectively, for parasagittal meningiomas following gross total resection [15]. For unresectable meningiomas, radiotherapy is an effective modality for local growth control. Systemic therapy for meningiomas remains largely experimental, reserved for recurrent/progressive disease refractory to surgery or radiotherapy, and no definitive standard of care has been established [10].

The diagnostic and therapeutic value of TRK in meningiomas remains unclear. Recent studies have highlighted the importance of standardized IHC markers in meningioma subtyping and risk stratification, with emerging data on the performance of targeted IHC panels in clinical neuropathology practice [16, 17, 18]. To characterize the expression profile and clinical relevance of TRK in meningiomas, we performed pan‐TRK, epithelial membrane antigen (EMA), progesterone receptor (PR), somatostatin receptor 2 (SSTR‐2), and S100 IHC staining on 50 meningioma specimens and 20 non‐meningioma CNS tumor specimens. This study aimed to delineate pan‐TRK expression patterns, genetic alteration status, and its potential role in differential diagnosis of meningiomas.

## Materials and methods

### Case selection

Seventy specimens of primary intracranial tumors were retrieved from the archives of the First Affiliated Hospital of China Medical University between January 2020 and December 2022. These included 50 meningiomas [21 World Health Organization (WHO) grade 1, 26 grade 2, and 3 grade 3, classified strictly according to the 2021 WHO Classification of Tumors of the Central Nervous System [19]] and 20 non‐meningioma tumors (9 solitary fibrous tumors, 6 hemangioblastomas, and 5 schwannomas). All specimens were formalin‐fixed, paraffin‐embedded (FFPE) surgical specimens. Clinicopathological characteristics, including patient age, gender, tumor location, WHO grade, and Ki‐67 proliferation index, were analyzed retrospectively. This study was approved by the Ethics Committee of the First Affiliated Hospital of China Medical University. Written informed consent was waived due to the retrospective nature of the study, in accordance with the committee's guidelines.

### Immunohistochemistry

Tissue specimens were formalin‐fixed, paraffin‐embedded, and cut into 4‐μm‐thick sections. Pan‐TRK IHC was performed using the rabbit recombinant monoclonal anti‐pan‐TRK antibody (clone EPR17341, catalog number: 790‐4576, Ventana Medical Systems, Tucson, AZ, USA), which targets a conserved C‐terminal epitope of TRK‐A, ‐B, and ‐C proteins (conserved across both wild‐type and chimeric variants). Additional IHC staining was performed using the following antibodies: anti‐EMA (clone E29, Dako, Glostrup, Denmark), anti‐PR (clone PgR 636, Dako), anti‐SSTR‐2 (clone UMB1, Abcam, Cambridge, UK), and anti‐S100 (clone EP32, Dako). All IHC assays were conducted on the automated BenchMark XT platform (Ventana Medical Systems, Tucson, AZ, USA) following the manufacturer's standardized protocol. The myenteric plexus ganglia of normal appendiceal tissue were used as positive controls and stained concurrently with each batch of samples. Negative controls were prepared by substituting the primary antibody with non‐immune rabbit IgG (Dako). A tumor was considered pan‐TRK‐positive if ≥1% of tumor cells displayed immunoreactivity of any intensity exceeding background staining, as validated for the EPR17341 clone in prior solid tumor studies [20, 21, 22]. According to previous literature, the critical threshold for Ki67 is set at 5% [23, 24, 25]. Various subcellular staining patterns (cytoplasmic, membranous, nuclear, and perinuclear) were deemed positive, as previously described. Staining intensity was scored on a 3‐point scale: 1 (weak), 2 (moderate), and 3 (strong). All stained sections were independently evaluated by two board‐certified neuropathologists blinded to the clinicopathological data. Discrepancies were resolved through consensus review using a multi‐head microscope.

### Fluorescence *in situ* hybridization (FISH)

FISH was performed to validate *NTRK gene* aberrations in all 12 pan‐TRK IHC‐positive meningioma specimens. Commercially available dual‐color break‐apart probes targeting NTRK1 (1q23.1, catalog number: 05 J74‐001), NTRK2 (9q21.33, catalog number: 03 N37‐020), and NTRK3 (15q25.3–q26.1, catalog number: 05 J75‐001) (Abbott Laboratories, Abbott Park, IL, USA) were used in accordance with the manufacturer's standard protocol. Briefly, FFPE sections were deparaffinized, pretreated with citrate buffer (pH 6.0) for antigen retrieval, and digested with proteinase K. Slides and probes were co‐denatured at 73 °C for 5 min, followed by probe hybridization at 37 °C overnight in a humidified chamber. After post‐hybridization washes, nuclei were counterstained with 4′,6‐diamidino‐2‐phenylindole (DAPI, D9542, Sigma‐Aldrich, USA) and mounted with anti‐fade medium.

FISH signals were analyzed using a fluorescence microscope (Olympus BX53, Tokyo, Japan) equipped with appropriate single‐bandpass filters. At least 100 non‐overlapping, intact tumor cell nuclei were counted per section by two independent observers. A positive result was defined as ≥10% of tumor cells showing split signals (separation of 5′ and 3′ probes by ≥2 signal diameters) or isolated 3′ signals, indicating NTRK gene rearrangement [9]. This threshold was selected based on prior validation studies demonstrating superior specificity for pathogenic NTRK fusions in solid tumors.

### Ethics statement

The study was conducted in accordance with the ethical standards set forth in the Declaration of Helsinki and was approved by the Institutional Review Board (IRB) of China Medical University. Informed consent was obtained from the patients for the use of their medical data for research and publication.

### Statistical analysis

Statistical analyses were conducted with SPSS 22.0 software. Categorical variables were presented as counts and percentages, and comparisons between groups were performed using the chi‐square test or Fisher's exact test (for cell counts <5). The correlation between pan‐TRK expression and clinicopathological characteristics was analyzed using Spearman's rank correlation coefficient. A two‐tailed *p* value <0.05 was deemed statistically significant.

## Results

### Immunohistochemical expression of pan‐TRK in meningiomas

**Figure 1:**
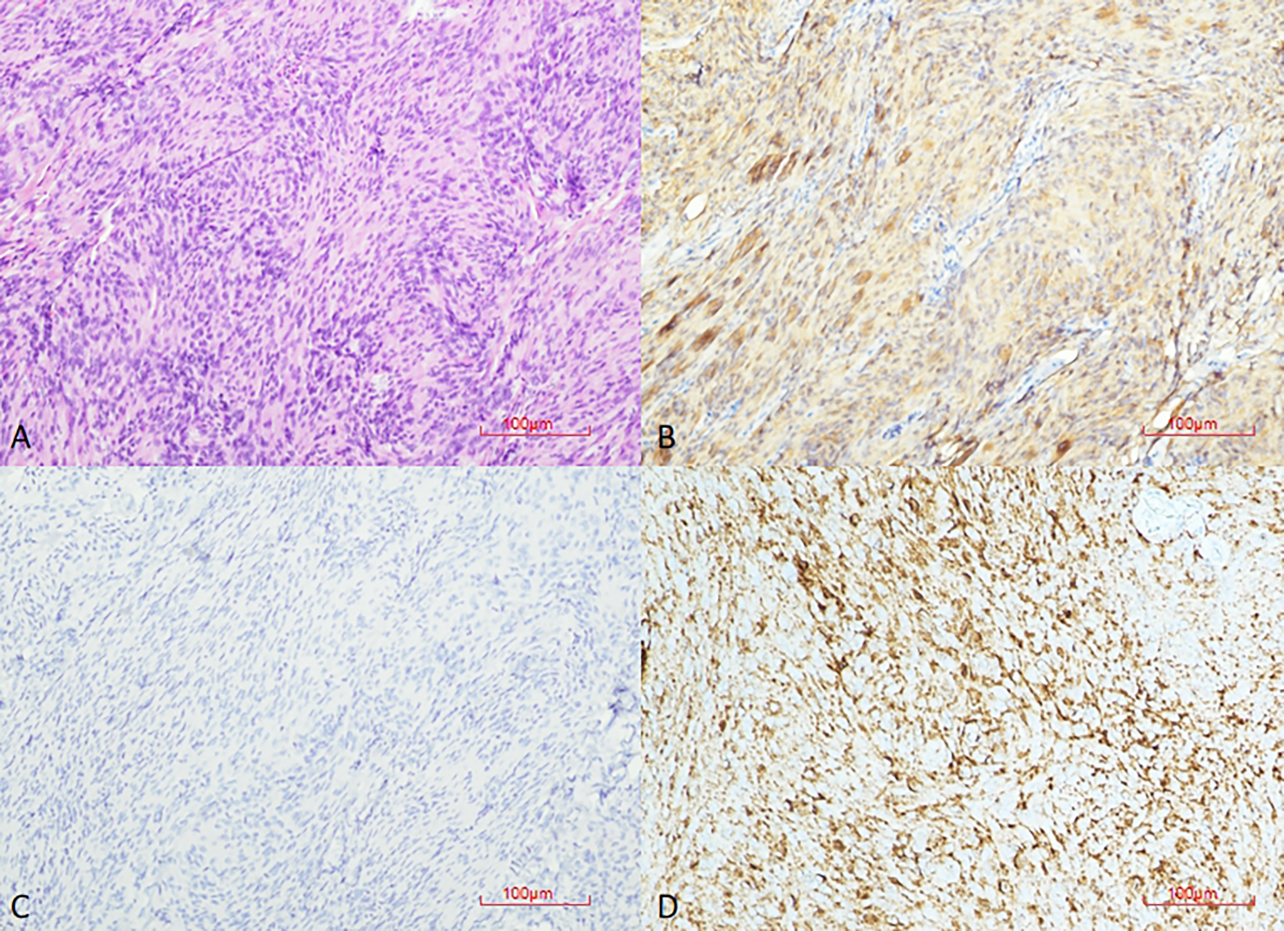
Representative histology and immunohistochemical staining of meningioma specimens. All images were captured at ×200 magnification; scale bar = 100 μm. (A) Hematoxylin and eosin (HE) staining of a typical WHO grade 1 meningothelial meningioma, showing whorled architecture and ovoid nuclei with indistinct cell borders. (B) Diffuse membranous and cytoplasmic immunoreactivity for SSTR‐2, supporting the diagnosis of meningioma. (C) Weak or absent S100 immunohistochemical staining in tumor cells, ruling out schwannoma in the differential diagnosis. (D) Strong diffuse cytoplasmic and nuclear pan‐TRK IHC staining (intensity score 3) in a WHO grade 3 anaplastic meningioma with confirmed NTRK3 rearrangement.

Pan‐TRK immunoreactivity was detected in 12 of 50 (24.0%) meningioma specimens. Representative hematoxylin and eosin (HE) and IHC images are shown in Figure 1. Among these positive cases, 8 (66.7%) exhibited weak staining (score 1), 3 (25.0%) moderate staining (score 2), and 1 (8.3%) strong staining (score 3). The subcellular staining pattern was predominantly cytoplasmic (*n* = 9, 75.0%), followed by combined cytoplasmic‐membranous (*n* = 2, 16.7%) and nuclear (*n* = 1, 8.3%). No perinuclear staining was observed.

Pan‐TRK positivity was significantly more frequent in high‐grade (WHO grade 2/3) meningiomas (7/29, 24.1%) than in low‐grade (WHO grade 1) tumors (3/21, 14.3%; *χ* ^2^ = 4.67, *p* = 0.031). When analyzed separately, the positivity rate was 5/26 (19.2%) in grade 2 meningiomas and 2/3 (66.7%) in grade 3 meningiomas. Of note, the grade 3 subgroup included only three cases, and these findings represent preliminary observations only, with no meaningful statistical inference possible for this grade stratum. In contrast, none of the 20 non‐meningioma CNS tumors (solitary fibrous tumors, hemangioblastomas, schwannomas) showed pan‐TRK immunoreactivity (0/20, 0%).

Spearman's rank correlation analysis demonstrated a significant positive correlation between pan‐TRK expression and WHO grade (*ρ* = 0.32, 95% CI 0.04–0.55, *p* = 0.023), as well as between pan‐TRK expression and Ki‐67 index (*ρ* = 0.35, 95% CI 0.08–0.58, *p* = 0.014). No significant correlations were observed between pan‐TRK expression and patient age (*p* = 0.628), gender (*p* = 0.415), or tumor location (*p* = 0.357) (Table 1).

**Table 1:** Correlation between pan‐TRK expression and clinicopathological characteristics of meningiomas

Characteristic including categories	Total (*n* = 50)	Pan‐TRK positive (*n* = 12, 24.0%)	Pan‐TRK negative (*n* = 38, 76.0%)	*p*	Spearman rho values	95% CI
Age, years				0.628	−0.031	(−0.306, 0.248)
<65	32	8 (25.0%)	24 (75.0%)			
≥65	18	4 (22.2%)	14 (77.8%)			
Gender				0.415	0.118	(−0.164, 0.385)
Male	17	3 (17.6%)	14 (82.4%)			
Female	33	9 (27.3%)	24 (72.7%)			
Tumor location				0.357	0.143	(−0.139, 0.411)
Skull base	23	4 (17.4%)	19 (82.6%)			
Convexity/parasagittal	27	8 (29.6%)	19 (70.4%)			
WHO grade				0.031*	0.302	(0.027, 0.543)^†^
1	21	3 (14.3%)	18 (85.7%)			
2	26	5 (19.2%)	21 (80.8%)			
3	3	2 (66.7%)	1 (33.3%)			
2/3 (high‐grade)	29	7 (24.1%)	22 (75.9%)			
Ki‐67 index, %				0.027*	0.347	(0.076, 0.582)
<5	35	5 (14.3%)	30 (85.7%)			
≥5	15	7 (46.7%)	8 (53.3%)			

* Statistically significant (*p* < 0.05).

^†^ CI (confidence interval) for grade included Gr1, 2, 3 for analysis; combined Gr2/3 category: for demonstration purposes only.

Sensitivity analysis using a more stringent cutoff (≥10% of tumor cells with staining intensity ≥2) yielded a pan‐TRK positivity rate of 4/50 (8.0%). All 4 cases were moderate‐to‐strong staining (score 2–3), and 2 of these 4 cases were FISH‐positive for *NTRK gene* aberrations, corresponding to a positive predictive value of 50% and specificity of 100% for fusion detection using this higher threshold.

### Correlation between TRK protein expression and molecular alterations

**Figure 2:**
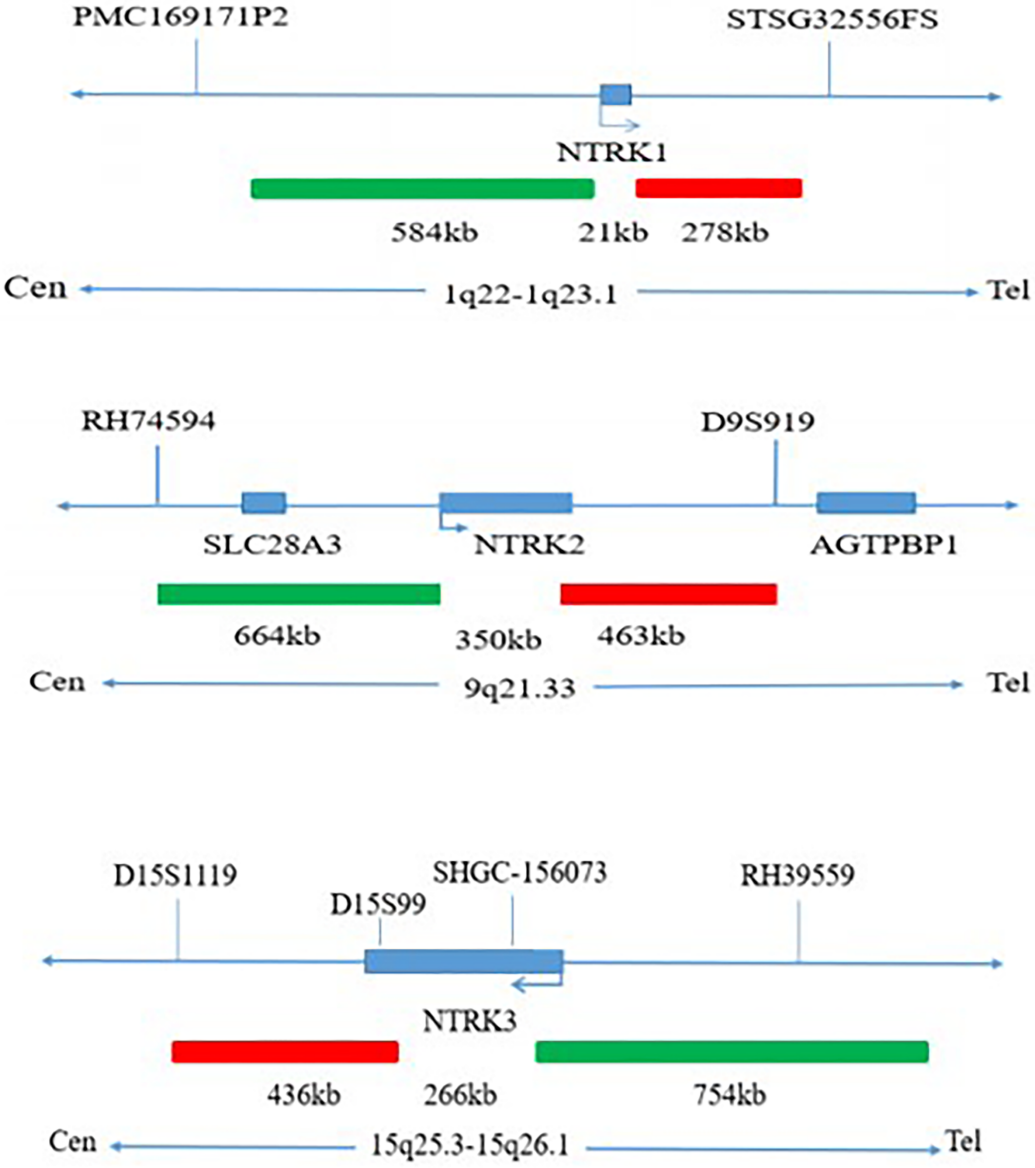
Schematic representation of NTRK break‐apart FISH probe design. Schematic illustration of the chromosomal loci and dual‐color break‐apart probe design for NTRK1 (1q22‐1q23.1), NTRK2 (9q21.33), and NTRK3 (15q25.3–15q26.1). For NTRK1 and NTRK2, green bars represent 5′ probes flanking the centromeric end of the NTRK coding region, and red bars represent 3′ probes flanking the telomeric end. For NTRK3 (transcribed in the opposite telomere‐to‐centromere direction), red bars represent 5′ probes flanking the centromeric end, and green bars represent 3′ probes flanking the telomeric end. The flanking sequence markers for each probe are labeled above the corresponding chromosomal regions, with the overall chromosomal orientation from centromere (left) to telomere (right). In non‐rearranged cells, the 5′ and 3′ probes co‐localize to produce a yellow fusion signal; in rearranged cells, the probes separate to produce distinct green and red signals.

**Figure 3:**
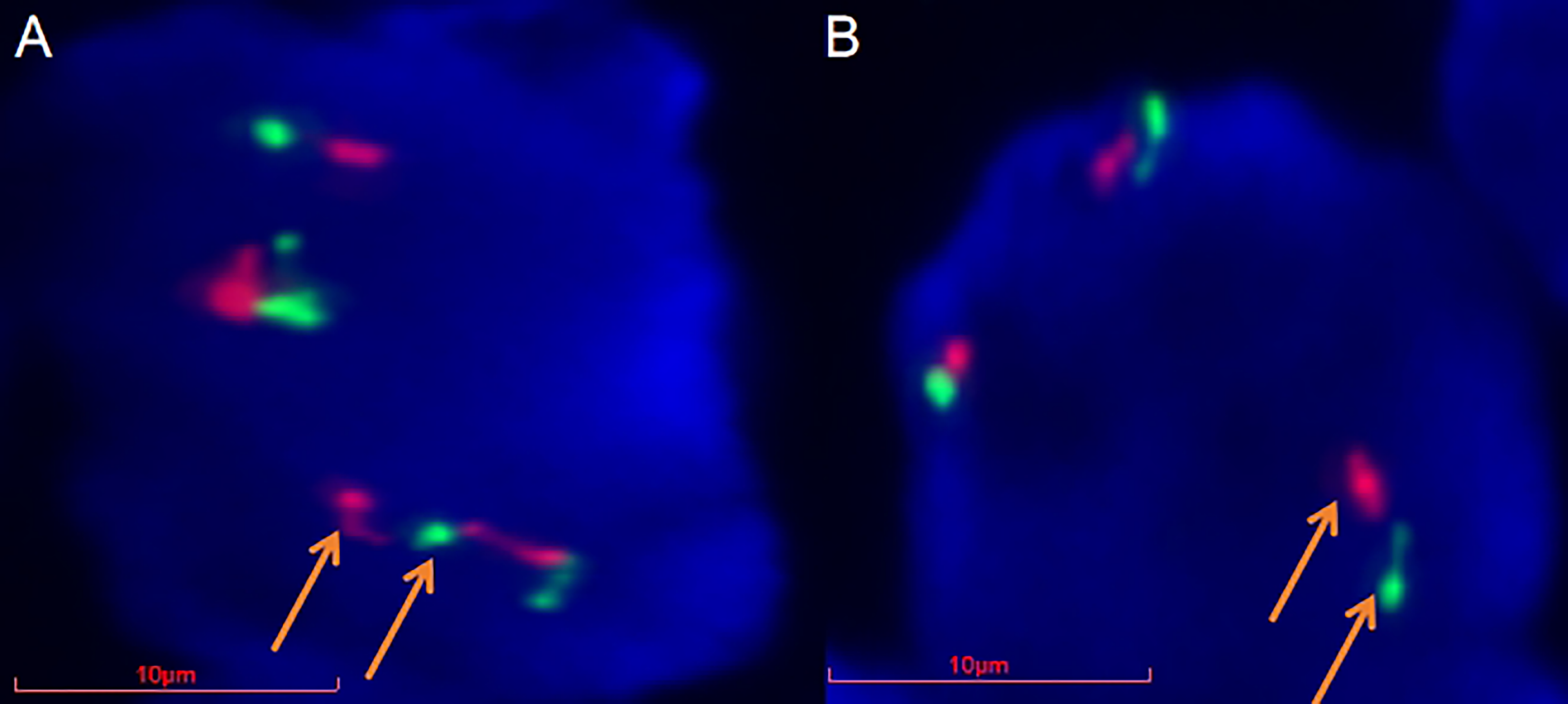
Representative FISH images of *NTRK gene* aberrations in meningioma specimens. All images were captured at ×1,000 oil immersion magnification; scale bar = 10 μm, with DAPI counterstain (blue) highlighting tumor cell nuclei. (A) NTRK3 break‐apart FISH showing split red (3′) and green (5′) signals (orange arrows) in a tumor cell nucleus, confirming NTRK3 gene aberrations. Inset: high‐magnification view of a representative positive nucleus with clear probe separation. (B) NTRK1 break‐apart FISH showing distinct split red and green signals (orange arrows) in tumor cell nuclei, confirming NTRK1 gene aberrations. Inset: high‐magnification view of a representative positive nucleus with isolated 3′ signal and co‐localized fusion signal in adjacent non‐rearranged nuclei.

FISH analysis was performed on all 12 pan‐TRK IHC‐positive meningiomas, with all three NTRK break‐apart probes applied to each case. The chromosomal loci targeted by the NTRK FISH probes are illustrated in Figure 2. *NTRK gene* aberrations were confirmed in 2 of 12 (16.7%) cases, both of which were WHO grade 3 anaplastic meningiomas. The remaining 10 (83.3%) pan‐TRK‐positive tumors showed no evidence of NTRK rearrangement by FISH. Notably, the single case with strong pan‐TRK staining (score 3) harbored an NTRK3 rearrangement (Figure 3A), while one case with moderate staining (score 2) harbored an NTRK1 rearrangement (Figure 3B). All 8 cases with weak‐staining cases (score 1) were FISH‐negative for *NTRK gene* aberrations. Detailed clinicopathological, IHC, and FISH data for all 12 pan‐TRK‐positive cases are summarized in Table 2.

**Table 2:** Detailed clinicopathological, IHC, and FISH data for 12 pan‐TRK‐positive meningioma cases

Case no.	WHO grade	Ki‐67 Index (%)	Pan‐TRK IHC intensity score	Staining pattern	FISH result
1	1	3	1	Cytoplasmic	Negative
2	1	4	1	Cytoplasmic	Negative
3	1	4	1	Cytoplasmic	Negative
4	2	6	1	Cytoplasmic	Negative
5	2	5	1	Cytoplasmic	Negative
6	2	7	1	Cytoplasmic	Negative
7	2	5	2	Cytoplasmic‐Membranous	Negative
8	2	8	2	Cytoplasmic	Negative
9	2	6	2	Cytoplasmic	Negative
10	3	18	2	Cytoplasmic‐Membranous	NTRK1 aberrations
11	3	22	3	Nuclear	NTRK3 aberrations
12	3	12	1	Cytoplasmic	Negative

## Discussion

While NTRK fusions are infrequent across all malignancies, they represent critical oncogenic drivers and diagnostic biomarkers in several tumor types [26], including mammary‐analog secretory carcinoma of the salivary gland (93–100% harboring ETV6‐NTRK3 fusions), secretory breast carcinoma (92% with ETV6‐NTRK3 fusions), infantile congenital fibrosarcoma (86–91% with ETV6‐NTRK3 fusions), and pediatric non‐brainstem high‐grade gliomas [27]. In primary CNS tumors, NTRK fusions occur in approximately 2% of adult cases (e.g., gliomas of all grades) and up to 5.3% of pediatric high‐grade gliomas and 2.5% of pediatric low‐grade gliomas [8]. These data highlight the potential utility of TRK‐targeted therapy in a subset of CNS tumors, underscoring the need for reliable screening strategies.

Immunohistochemistry is an efficient screening tool for NTRK fusions in most tumor types; however, its specificity in CNS tumors is notably suboptimal due to the physiological expression of NTRK in normal neural tissues [28]. For example, Solomon *et al*. reported a specificity of only 20.8% for gliomas [29], rendering pan‐TRK IHC alone insufficient for definitive diagnosis. Significant false‐positive IHC results have also been observed in tumors with smooth muscle or neuroendocrine differentiation and small round cell tumors. Notably, false‐positive staining is typically restricted to the cytoplasm and/or cell membrane, with no nuclear localization – a finding consistent with our observation that only 1 of 12 pan‐TRK‐positive meningiomas showed nuclear staining (and this case was FISH‐positive for NTRK3 rearrangement) [28]. This suggests that nuclear staining may be a more specific indicator of pathogenic NTRK fusion, though further validation in larger cohorts is needed.

Meningiomas are categorized into 15 subtypes spanning three WHO grades, with distinct clinical behaviors and molecular profiles [30]. WHO grade 1 meningiomas are benign, slow‐growing neoplasms accounting for approximately 80% of all cases, with meningothelial and fibroblastic subtypes being the most common. WHO grade 2 meningiomas (15–20% of cases) are defined by increased mitotic count, significant brain invasion, cytological pleomorphism, and/or tumoral necrosis. Anaplastic meningiomas (WHO grade 3) are highly aggressive malignant neoplasms, accounting for only 1–2% of cases, with poor clinical outcomes [13, 31, 32]. The most frequent cytogenetic aberration in meningiomas involves NF2 gene mutations, observed in approximately 60% of sporadic cases [33]. Genetic aberrations in non‐NF2 meningiomas encompass mutations in the PI3K‐AKT signaling pathway (AKT1, mTOR, PTEN, PIK3CA, PIK3R1), Hedgehog signaling pathway (SMO, SUFU, PRKAR1A, PTCH1/2), chromatin remodeling complexes (SMARCB1, SMARCE1, ARID1A, PBRM1), and other genes (TRAF7, KLF4, BAP1, POLR2A, DMD, etc.) [13, 34, 35, 36, 37].

Meningiomas arising at different anatomical sites exhibit distinct embryonic lineages: skull base meninges derive from the mesoderm, while convexity (telencephalic) meninges originate from the neural crest [38]. Accordingly, these tumors may harbor distinct histological subtypes and driver gene profiles. For example, convexity meningiomas are predominantly fibroblastic or transitional subtypes, often associated with NF2 mutations [39], and the proportion of WHO grade 2/3 tumors is significantly higher in convexity or parasagittal locations than in skull base grade 1 meningiomas. Skull base meningiomas are mainly meningothelial, secretory, or microcystic subtypes, frequently harboring mutations in AKT1, SMO, PIK3CA, TRAF7, POLR2A, and KLF4 [13, 40, 41]. Spinal meningiomas and clear cell meningiomas often exhibit SMARCE1 deletion mutations [42, 43]. The 2021 WHO Classification of Tumors of the CNS (5th edition) for the first time incorporated molecular genetic signatures into the meningioma classification framework: concurrent TRAF7 and KLF4 mutations serve as molecular markers for secretory meningiomas, SMARCE1 mutations are characteristic of clear cell meningiomas, and BAP1 mutations are associated with rhabdoid meningiomas [43]. In terms of grading, TERT promoter mutations and/or homozygous deletion of CDKN2A/B are sufficient to warrant a diagnosis of WHO grade 3 anaplastic meningiomas, regardless of anaplastic histological features. Additionally, loss of H3K27me3 is a well‐established predictor of unfavorable prognosis.

Our study demonstrates that pan‐TRK is expressed in 24.0% of meningiomas, with higher positivity rates in high‐grade tumors and those with elevated Ki‐67 indices. Previous studies have confirmed that Ki‐67 can predict prognosis and improve the diagnostic accuracy of meningiomas [17, 44]. We confirm that the threshold of Ki‐67 indices was pre‐specified based on prior published literature establishing 5% as a clinically relevant stratifier of meningioma proliferative activity and recurrence risk [23, 24, 25]. Notably, none of the 20 non‐meningioma CNS tumors in our cohort showed pan‐TRK immunoreactivity. However, given the small size and heterogeneity of this control group, this finding should be interpreted as preliminary and hypothesis‐generating only and does not establish pan‐TRK IHC as a definitive diagnostic marker for differentiating meningiomas from other CNS tumors. Larger cohorts including a broader spectrum of meningeal and CNS neoplasms are required to validate the differential diagnostic utility of pan‐TRK IHC in this context.

Only 16.7% of pan‐TRK‐positive meningiomas harbored *NTRK gene* aberrations in our study, indicating that most pan‐TRK expression in meningiomas is not driven by fusion events. This is consistent with previous reports of low NTRK fusion rates in meningiomas [18] and suggests that pan‐TRK IHC alone cannot reliably identify fusion‐positive cases. Instead, pan‐TRK IHC may serve as a preliminary screening tool, with positive results requiring confirmation by FISH or next‐generation sequencing (NGS) – the gold standard for detecting NTRK rearrangements [9]. Of note, our study did not perform NGS, which is a limitation, as FISH may fail to detect rare NTRK fusion variants, intragenic rearrangements, or alternative splicing events that may be clinically relevant.

The clinical significance of pan‐TRK expression in meningiomas without NTRK rearrangements remains unclear. It is possible that non‐fusion‐mediated pan‐TRK overexpression contributes to meningioma progression through alternative signaling mechanisms, though this requires further investigation. Additionally, our finding of higher pan‐TRK positivity in high‐grade meningiomas suggests a potential association with aggressive behavior, which warrants validation in larger, prospective cohorts. In certain malignant tumors, NTRK fusion may indicate higher invasiveness and recurrence risk [45, 46]. Importantly, both NTRK‐rearranged cases in our study were restricted to the three grade 3 meningiomas; however, given the extremely small size of this subgroup, no meaningful statistical or clinical conclusions can be drawn from this observation, which should be considered strictly preliminary.

This study has several limitations. First, the sample size is relatively small, particularly for grade 3 meningiomas (*n* = 3), which may limit the generalizability of our findings. Second, we did not perform NGS to assess for rare NTRK variants or alternative genomic events that may not be detected by FISH. Third, the retrospective design precluded analysis of the prognostic impact of pan‐TRK expression or NTRK aberrations on patient survival or recurrence. Future studies with larger multi‐institutional cohorts, long‐term clinical follow‐up, and comprehensive molecular profiling are needed to clarify the role of TRK in meningioma pathogenesis and therapy.

In conclusion, pan‐TRK is frequently expressed in meningiomas, particularly in high‐grade and proliferative tumors, but *NTRK gene* aberrations are rare. Pan‐TRK IHC may have preliminary utility in the differential diagnosis of meningiomas from other CNS tumors, but FISH or NGS confirmation is mandatory to identify true fusion‐positive cases eligible for TRK‐targeted therapy. Further research is needed to elucidate the biological function of non‐fusion‐mediated pan‐TRK overexpression in meningiomas and its potential as a prognostic biomarker or therapeutic target.

## Author contributions statement

YZ, MS and XL designed the research. YZ, MS, WL, ZW and XL conducted the search. MS, YZ, HaX, HoX and YS had full access to the study data and carried out all analyses. All the authors contributed to the manuscript writing, read and approved the final manuscript.

## Data Availability

For further information or data requests, contact the corresponding author on reasonable request.
